# User Requirements in Developing a Novel Dietary Assessment Tool for Children: Mixed Methods Study

**DOI:** 10.2196/47850

**Published:** 2024-02-01

**Authors:** Zoë van der Heijden, Femke de Gooijer, Guido Camps, Desiree Lucassen, Edith Feskens, Marlou Lasschuijt, Elske Brouwer-Brolsma

**Affiliations:** 1 Division of Human Nutrition and Health Wageningen University & Research Wageningen Netherlands

**Keywords:** diet, children, dietary assessment, recall, technological innovation, mobile health, mHealth, mobile phone

## Abstract

**Background:**

The prevalence of childhood obesity and comorbidities is rising alarmingly, and diet is an important modifiable determinant. Numerous dietary interventions in children have been developed to reduce childhood obesity and overweight rates, but their long-term effects are unsatisfactory. Stakeholders call for more personalized approaches, which require detailed dietary intake data. In the case of primary school children, caregivers are key to providing such dietary information. However, as school-aged children are not under the full supervision of one specific caregiver anymore, data are likely to be biased. Recent technological advancements provide opportunities for the role of children themselves, which would serve the overall quality of the obtained dietary data.

**Objective:**

This study aims to conduct a child-centered exploratory sequential mixed methods study to identify user requirements for a dietary assessment tool for children aged 5 to 6 years.

**Methods:**

Formative, nonsystematic narrative literature research was undertaken to delineate initial user requirements and inform prototype ideation in an expert panel workshop (n=11). This yielded 3 prototype dietary assessment tools: FoodBear (tangible piggy bank), myBear (smartphone or tablet app), and FoodCam (physical camera). All 3 prototypes were tested for usability by means of a usability task (video analyses) and user experience (This or That method) among 14 Dutch children aged 5 to 6 years (n=8, 57% boys and n=6, 43% girls).

**Results:**

Most children were able to complete FoodBear’s (11/14, 79%), myBear’s (10/14, 71%), and FoodCam’s (9/14, 64%) usability tasks, but all children required assistance (14/14, 100%) and most of the children encountered usability problems (13/14, 93%). Usability issues were related to food group categorization and recognition, frustrations owing to unsatisfactory functioning of (parts) of the prototypes, recall of food products, and the distinction between eating moments. No short-term differences in product preference between the 3 prototypes were observed, but autonomy, challenge, gaming elements, being tablet based, appearance, social elements, and time frame were identified as determinants of liking the product.

**Conclusions:**

Our results suggest that children can play a complementary role in dietary data collection to enhance the data collected by their parents. Incorporation of a training program, auditory or visual prompts, reminders and feedback, a user-friendly and intuitive interaction design, child-friendly food groups or icons, and room for children’s autonomy were identified as requirements for the future development of a novel and usable dietary assessment tool for children aged 5 to 6 years. Our findings can serve as valuable guidance for ongoing innovations in the field of children’s dietary assessment and the provision of personalized dietary support.

## Introduction

In the Netherlands, childhood overweight and obesity have reached an alarming prevalence of 15% in 2021, indicating a pressing public health concern [1]. Childhood obesity has a major impact on psychological health and later-life risks of developing noncommunicable diseases, mortality, and morbidity [2,3]. As a healthy diet is known to play a vital role in the prevention of obesity [4], interventions encouraging children toward healthier food choices receive high priority globally. However, the long-lasting effects of past and ongoing dietary interventions are rather limited, mainly related to their “one-size-fits-all” approach, which calls for more personalized interventions [5].

Personalized dietary interventions require accurate individual-level dietary assessment and monitoring [6] to facilitate realistic personalized dietary feedback. However, dietary assessment methods in young children are extremely challenging owing to their limited literacy, writing skills, food knowledge, and interest [7]. Consequently, caregivers currently serve as the primary sources of (surrogate) dietary information for their children. However, as primary school children gain independence in their food choices, the likelihood of misreporting increases [8]. Recent technological advancements now provide opportunities for a role for children themselves in the dietary assessment, which may serve the overall obtained dietary data quality.

Accordingly, there is growing interest in the development of innovative tools to assess dietary intake in children, with a particular emphasis on more effective and engaging technology-based solutions [9,10]. However, most of the developed tools thus far lack proper validation and are not tailored to the Dutch context, including Dutch food databases [11,12]. Country-specific dietary assessment tools are essential to accurately capture dietary information while considering cultural, regional, and nutritional differences. Moreover, given children’s rapid cognitive development, there is also a need for dietary assessment tools that align with their age-specific developmental stages. Current research has mostly focused on tools for children aged ≥8 years [11], as children tend to be better at independently reporting their food intake from this age onward [7]. However, considering the high level of technology readiness in today’s generation of children and the continuously changing technological possibilities, it is worth exploring the development of dietary assessment tools for younger children.

A “child-centered approach,” which places the user in the center of the design and development process, can effectively address young children’s age-specific cognitive needs for innovative dietary assessment. This approach enables researchers to understand the context, needs, and preferences of the tool’s intended end users [13,14] by engaging children in identifying challenges and finding solutions [15]. As a result, a child-centered approach can enhance design outcomes, improve user experience [16], and potentially improve data collection procedures and accuracy. In the decades marked by an increasing prevalence of childhood health issues, understanding the dietary behaviors and needs of young children is vital for informing effective interventions.

Therefore, as a first step, this study aimed to reveal user requirements for a novel child-friendly food intake registration tool designed for Dutch children aged 5 to 6 years. In pursuit of this goal, we developed and evaluated 3 distinct prototypes specifically created for children aged 5 to 6 years while considering age-specific cognitive and developmental characteristics to serve as valuable guidance for advancing the field of dietary assessment tools for children across a broader age range.

## Methods

**Figure 1:**
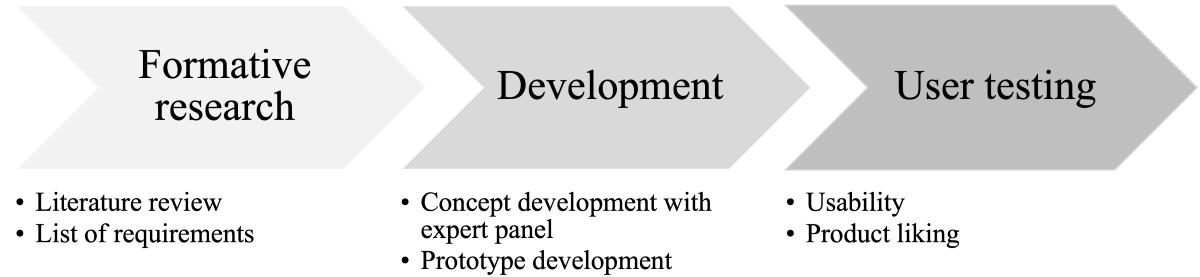
Flowchart illustrating the design process of the dietary assessment tool prototypes FoodBear, myBear, and FoodCam.

This study applied an exploratory sequential mixed methods study design, combining qualitative and quantitative measures [17]. Phases included a formative research phase (qualitative), a developmental phase (qualitative), and an evaluation phase (mixed methods, but with qualitative emphasis; Figure 1).

### Formative Research

We performed a nonsystematic narrative literature research to identify existing dietary intake assessment tools for young children, their validity, and age-specific developmental considerations. This information was then used in an expert workshop to probe prototype idea generation. This nonsystematic search followed an abductive approach [18], focusing on extracting valuable insights to inform the design process effectively (eg, formulation of a list of user requirements and wishes for designing a dietary assessment tool for children aged 5-6 years; Textbox 1), rather than aiming for an exhaustive review of the available literature. The search query included a combination of the following Medical Subject Headings terms: *dietary assessment*, food intake, nutritional assessment, child centered design, child*, kid*, preschool*, child computer interaction, and *eHealth*. To our knowledge, this study is the first to focus on self-reported tool development among children aged 5 to 6 years. Therefore, our requirements are based on heterogeneous literature, including studies related to design for children [19-21], dietary assessment tools for older children outside the Netherlands [22-27], or child development [7,28]. In the context of this study, requirements were defined as being vital for usability and wishes as being desirable for enhancing usability and motivation among children. Our requirements were assessed in terms of perceived importance (ranging from 1 to 5) based on close consultation and consensus within our research team (Textbox 1). In the weighted decision matrix (WDM), decisions were rated on a scale of 1 to 5, with 1 indicating low importance and 5 signifying high importance for successful use.

**List of user requirements for a novel dietary assessment tool for children resulting from formative research and their importance (score ranging from 1 to 5). The list includes aspects that the dietary assessment tool should have (ie, requirements) or could have (ie, wishes).**

**Requirements (importance score)**

- Collect accurate and useful data on dietary intake in children (importance score 5) [7,22-26]
- Be understandable (ie, simple, easy-to-use, and intuitive; importance score 5) [26]
- Be fast paced (ie, completed in a short time; importance score 4) [26,28]
- Give feedback and context-specific help (eg, auditory or visual; importance score 3) [19,20]
- Be motivating and encouraging to use (importance score 3) [19,26,27]
- Be social (importance score 3) [19]
- Be challenging (importance score 3) [19]

**Wishes**

- Incorporate photography [22,27]
- Incorporate an avatar [21,25,26]
- Incorporate gamification [21,26]
- Include a storyline [21,25,26]
- Include rewards [21]
- Incorporate learning and/or repetitive elements [19]

### Development

#### Idea Generation

Concepts for dietary assessment tools were developed through a 1-hour web-based expert panel workshop hosting nutrition (n=4), design (n=4), behavior (n=1), and technology researchers (n=2). Experts were carefully selected from various universities in the Netherlands and came together on the web-based Miro whiteboard platform. The workshop unfolded in 3 key stages. First, the experts immersed themselves in the world of our target audience by engaging with emotional image prompts. Next, experts were presented with our comprehensive list of requirements, as detailed in Textbox 1, and tasked with generating prototype ideas that could address these requirements. In the third and final phase of the workshop, the experts collaborated in pairs to refine these ideas and transform them into feasible prototypes. The results of this collaborative effort produced a wide range of innovative concepts, including a food piggy bank, food camera, digital plate, Tamagotchi, smartwatch, and a food diary.

#### Prototype Development

**Figure 2:**
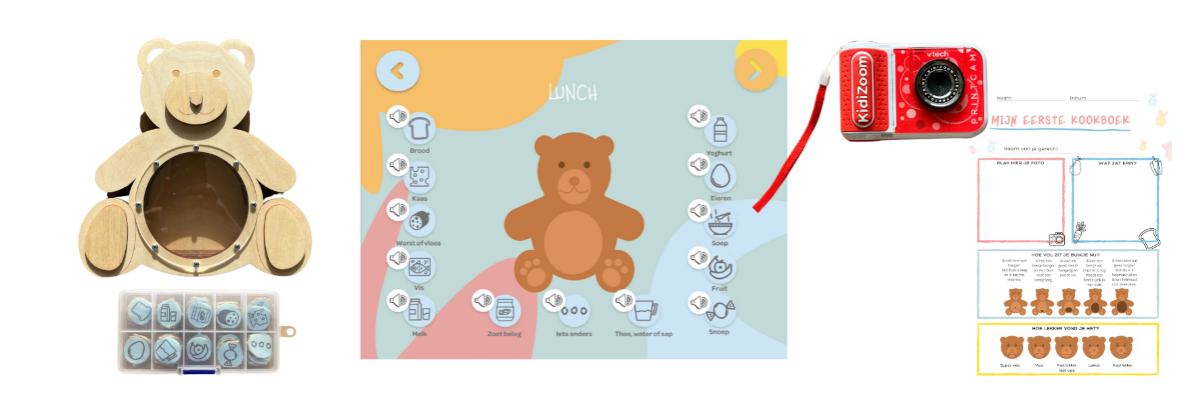
From left to right, FoodBear, myBear, and FoodCam. myBear (in Dutch) shows the entry screen for lunch, where children can report their intake during lunch based on the food groups depicted in Textbox 1.

The results of the expert panel workshop were scored and evaluated against the list of requirements and subsequently multiplied by their importance (ranging from 1 to 5) in a WDM [18] (Multimedia Appendix 1), a decision-making tool that can be used to evaluate a set of options against critical factors and compare design concepts based on the overall value of each design concept. Three researchers from Wageningen University and Research (WUR) completed the WDM individually to ensure objectivity. The 3 concepts were considered to align most closely with the requirements and subsequently further advanced, which resulted in the 3 functional prototypes, FoodBear (average WDM score: 144.1), myBear (average WDM score: 148.4), and FoodCam (average WDM score: 144.7; Figure 2; Multimedia Appendix 2).

FoodBear can be used as a food recall or food record [9] and serves as a physical eating buddy in the shape of a bear. FoodBear can be fed with coins that match the foods consumed by the child (Table 1). A coin represents 1 item of the food group eaten (eg, 1 slice of bread) and children are challenged to put the number of coins corresponding to the number of items eaten in the bear’s belly as an estimate of portion size. This allows the assessment of food group diversity and provides a rough estimate of dietary intake.

**Table 1:** Included food groups in myBear and FoodBear and their contribution to the lunch of Dutch children aged 5 to 6 years [24].

Food group	Contribution to lunch (%)	Child-friendly categories in prototype
Cereals and grain products	43	Bread
Eggs	28	Eggs
Milk products	26	Milk, yogurt, and cheese
Soups, broth	23	Soup
Meat and meat products	20	Sausage or meat
Sugar and confectionery	15	Sweet toppings and candy
Fruits, nuts, and olives	14	Fruit
Different	13	Something different
Fish, crustaceans, and shellfish	12	Fish

myBear can be used as a food recall or food record [9] but in the form of a tablet-based app. The user interface design was developed with Adobe XD software, and the prototype app can be displayed on a smartphone or tablet. Children feed the bear with the same foods as they ate themselves. On the home screen of the app, children select the food groups they consumed, which then appear in the belly of myBear. The child uses plus and minus buttons to indicate the quantities of items from the food group they have consumed. Children receive a sticker on a digital sticker sheet after completing all entries. Similar to FoodBear, myBear can be used to provide a rough estimate of dietary intake and to track food group diversity.

FoodCam is based on the food record methodology [9] and consists of a camera and a “cookbook template.” The camera is specifically designed for children, and for this specific purpose, it was used to take pictures of food items. As the camera immediately prints the captured photo, it also provides immediate feedback to the child. Subsequently, the printed photos can be used by children to create their own cookbook, which offers the opportunity for children to draw and express their creativity. FoodCam can be used to assess daily food intake. FoodCam is accompanied by a cookbook stencil (in Dutch) on which children can put a picture of their lunch, draw it, indicate how full they feel [29], and how much they enjoyed it [30].

#### Prototype Content

The first prototypes were developed to assess lunchtime and focused on the most frequently consumed foods during lunchtime by young Dutch children according to the Dutch Food Consumption Survey [31] (Table 1). Only food groups that contributed for >10% to children’s lunch were included.

### Recruitment

#### Overview

We recruited 14 Dutch (n=6, 43% girls and n=8, 57% boys) children aged 5 to 6 years through purposive sampling, as 10 (±2) participants were considered sufficient for usability evaluation [32]. Data saturation was assumed to be acquired within 6 to 12 sessions [33], which in this study was reached after 11 sessions. Children were recruited via colleagues within the Division of Human Nutrition and Health at WUR and through personal networks.

#### User Testing

The functional prototypes were evaluated on usability and user experience by combining qualitative and quantitative measures [17]. To make the child feel comfortable, the researcher visited the child at home in the presence of a parent or caregiver. Before the start of the session, the parent or caregiver was instructed to introduce the researcher as a toy inventor and that their child would act as an assistant inventor. Moreover, parents were instructed to interfere as little as possible during the user tests. The entire procedure took approximately 30 minutes and took place after lunch, between 1:30 PM and 3:00 PM. The 30-minute time frame was selected to align with the attention span of young children. We conducted 3 streamlined usability tasks (lasting a maximum of 140 seconds) and a user experience task and interview within this time frame to minimize any potential loss of interest or fatigue among participating children. To ensure that all tasks would fit within our proposed time frame, the procedures were piloted twice. All parents completed a demographic questionnaire on the child’s age (y),gender (boys or girls), number of siblings, siblings’ age (y), interactive screen time (h/d), foods and portion sizes eaten during lunch, and time of lunch. Parental lunch dietary intake data gathered in the questionnaire were used as the criterion to assess successful recall by the children, instead of more objective direct lunch observation, to be able to create a study environment that is comfortable and engaging for children. Testing order for FoodBear and myBear was alternated across participants. To assess the usability of FoodCam, parents were instructed to prepare a duplicate (ie, an “identical meal to what their child had eaten for lunch on the day of testing”) of the children’s previously consumed lunch during the test. This prototype was always tested last because FoodCam is the only prototype that does not rely on children memorizing their lunch.

The procedure consisted of 3 usability tests (steps 3, 5, and 6; Textbox 2) and a user experience test (steps 7-9; Textbox 2). To assess usability, children performed a task with every prototype while measuring the completion rate and task completion time. Completion rate was defined as the proportion of children that successfully completed the usability tasks, and completion time was defined as the time needed to complete the task. The time required for the researcher to explain or draw attention to the task was subtracted and the number of interruptions required to complete the tasks were registered. Behavioral observations and field notes were evaluated to identify the usability issues. A detailed description of the study procedures is provided in Textbox 2.

**Within-participant procedures consisting of 9 steps (maximum 30 min in total). Steps 3 and 5 are alternated across participants.**

**Step 1: Introduction**

- The researcher engaged in a small talk with the child to build trust. Study procedures were explained in a child-friendly manner, and it was emphasized that the child could say anything.

**Step 2: Lunch recall**

- As the usability task of myBear and FoodBear required recall of the lunch, the child was asked to do this before starting these tasks. Lunch was considered as being correctly recalled when it resembled the lunch written down in the questionnaire by caregivers, in terms of food items and numbers. If the child was unable to recall his or her lunch independently, standardized help questions were asked: (1) “Did you eat bread?” (2) “How much bread did you eat?” (3) “What kind of topping did you eat? Cheese, meat or something sweet?” (4) “How much bread did you eat with this topping?” If the child answered a question with “no,” the following questions were prepared: (5) “Did you eat a salad, pasta or rice for lunch?” (6) “Did you drink something with your lunch? Milk, tea, or water?” (7) “Did you eat anything else, such as candy, fruit or soup?” The number of questions required was noted.

**Step 3: Usability task: myBear**

- The child was asked to provide myBear with the same foods as recalled in step 2 or 4. The session started by clicking the “lunch” button (ie, as one of 5 different eating moments), which started the time measurement. The time measurement ended once the last food item was entered in myBear. The assignment was completed when all food groups and amounts were correctly entered.

**Step 4: Lunch recall**

- To mitigate potential effects arising from the passage of time between recalling the lunch and subsequent assessment of prototype’s usability, the child was asked to recall the lunch again.

**Step 5: Usability task: FoodBear**

- The child was asked to give the same lunch to FoodBear as recalled in step 2 or 4. The time measurement started once the researcher asked the child to start feeding FoodBear and ended when the child put the last coin into its belly. The assignment was completed when all food groups and amounts were correctly entered.

**Step 6: Usability task: FoodCam**

- The child was asked to take a picture of his or her (duplicate) lunch with FoodCam. Time measurement started once the researcher handed over the camera to the child and ended when the child took the picture. The assignment was completed when the (1) photo was sharp and (2) included all consumed foods in a recognizable way. To assure objectivity, photos were assessed by 3 researchers.

**Step 7: This or That method**

- As a response to 5 “This or That” questions, children indicated which prototype they liked best [34]. The original This or That method uses pairwise comparison, but this study compared 3 prototypes. One of the original questions was considered irrelevant and excluded: “Which of these three would you most like to take home?” The following This or That questions were included: “Which of these three was most fun?” “Which of these three was a bit stupid?” “Which of these three was a little boring?” “Show me which of these three you would like to play again?” “Show me which of these three you would like to receive as a gift?” Children could indicate more than 1 prototype but were not told beforehand to facilitate decision-making.

**Step 8: Reward**

- The child received a biscuit and a stamp set to express gratitude for time investment and participation in the study.

**Step 9: Behavioral choice selection**

- The researcher told the child “that there was some time left,” and that he or she could select a prototype to play with again. The researcher ensured that the child ate the biscuit first to prevent the child from automatically choosing the prototype they played with last.

### Data Analysis

#### Usability

All sessions were audio- and video-recorded and transcribed verbatim. The researcher watched the videos and documented the examples of interest. Using the qualitative data analysis software ATLAS.ti (ATLAS.ti Scientific Software Development GmbH), examples of interest were grouped into themes to identify the most important usability issues, by means of using a reflexive and inductive approach, allowing for the emergence of unexpected insights, and understanding of prototype usability. Specific attention was paid to behaviors that hindered the completion of the usability tasks. As this study was exploratory in nature, our goal was to generate a foundation for further research. Therefore, data were coded by a single coder to gain a deeper understanding of the research objectives and context. To determine usability task effectivity and efficiency for each prototype, average time and corresponding SDs were calculated for the usability tasks. By integrating qualitative and quantitative usability in the discussion and interpreting our results, we aimed to provide a more comprehensive assessment of the prototypes’ usability among the target group.

#### User Experience

The quantitative results for This or That method were coded dichotomously for each of the 5 questions. A preference was scored 1 in case of the 3 positive questions. In contrast, a preference was scored −1 in case of the 2 negative questions. Consequently, the total score for the 3 prototypes ranged from a minimum of −2 to a maximum of 3, for which a mean score and corresponding SD were calculated. One-way ANOVA was performed to test for significant differences using SPSS Statistics (version 25; IBM Corp). A *P* value of ≤.05 was considered statistically significant. Furthermore, qualitative data were analyzed by using a combination of deductive and inductive thematic coding in ATLAS.ti. Qualitative and quantitative data were integrated to gain a deeper understanding of prototype user experience.

### Ethical Considerations

All parents provided written informed consent and children gave their verbal consent. When the child did not fully understand the study procedures, these were explained again. Participation was voluntary and participants could withdraw from the study at any time, without stating a reason. The study protocol was reviewed and deemed not subject to the Medical Research Involving Human Subjects Act (2021-13199). Subsequently, the protocol was reviewed and approved by the Social Sciences Ethics Committee of the WUR. The organization conducting this study established procedures for data management and data protection. Participants were not financially compensated for participating in this study, but children received a small gift as a thank you (a cookie and a stamp set).

## Results

### Sample Characteristics

Table 2 presents the descriptive characteristics of the study sample. A total of 14 children participated in the study and evaluated the 3 prototypes. Most children had at least 1 highly educated parent (12/14, 86%) and actively used technology for ≤1 hour per day (11/14, 79%). Moreover, most of the children had no older siblings (11/14, 79%).

**Table 2:** Sociodemographic participant characteristics across usability and user experience tasks (N=14).

Characteristics		Values
**Gender, n (%)**		
	Boys	8 (57)
	Girls	6 (43)
**Age (y), mean (SD)**		5.9 (0.8)
	Boys	6.0 (0.6)
	Girls	5.6 (1.0)
**Older siblings, n (%)**		
	Yes	3 (21)
	No	11 (79)
**Interactive screen time^a^, n (%)**		
	≤1 h daily	11 (79)
	2 h daily	3 (21)
	3 h daily	0 (0)
**Education level of caregiver or caregivers^b^, n (%)**		
	No highly educated caregiver	2 (14)
	1 highly educated caregiver	2 (14)
	2 highly educated caregivers	10 (72)

^a^Interactive screen time use is defined as the time a child spends actively interacting with a device (eg, tablet, PC, or smartphone).

^b^“Highly educated” is defined as having completed a degree at a university or a university of applied sciences.

### Usability Testing

#### Quantitative Results

Of 14 children, 10 (71%) correctly recalled their lunch; half of the boys (4/8, 50%) and all the girls (6/6, 100%) succeeded in recalling their lunch. None of the successful children were able to do so without the assistance of standardized recall questions. Overall, 1 (7%) child needed the maximum number of 5 recall questions and 4 (40%) children needed one recall question; successful children needed an average of 2.2 (SD 1.4) recall questions. FoodBear’s usability task had the highest completion rate (n=11, 79%), followed by myBear (n=10, 71%) and FoodCam (n=19, 64%). The mean completion time (s) was faster and the number of interruptions (n) was lower for FoodCam (mean 9, SD 6; n=0.9) followed by myBear (mean 51, SD 17; n=3.3), and FoodBear (mean 65, SD 43; n=3.9; Table 3). Children who failed to complete either FoodBear’s (n=3, 21%) or myBear’s usability task (n=4, 29%) were all boys, but 1 (20%) girl failed to complete FoodCam’s usability task (n=5, 36%). Among these children, approximately all children had no highly educated parents (100%) or one highly educated parent (50%). When visually inspecting our data, no differences in age, interactive screen time (≤1 hour per day), or having older siblings (no) were observed across the children who did not complete their tasks. Finally, the order in which FoodBear and myBear were tested alternated. Children who successfully completed both FoodBear’s and myBear’s usability task (n=10, 71%) performed their second task on average 26.1 (SD 27.1) seconds faster.

**Table 3:** Descriptive data (task effectivity and task efficiency) of usability tasks of FoodBear, myBear, and FoodCam.

Task	Completion rate, n (%)	Completion time (seconds), mean (SD; range)	Interruptions for help, mean (SD)
FoodBear: “Give FoodBear the same lunch as you ate”	11 (79)	65 (43; 16-140)	4 (4)
myBear: “Give myBear the same lunch as you ate”	10 (71)	51 (17; 15-70)	3 (2)
FoodCam: “Photograph your lunch with FoodCam”	9 (64)	9 (6; 3-24)	1 (1)

#### Observational Results

**Figure 3:**
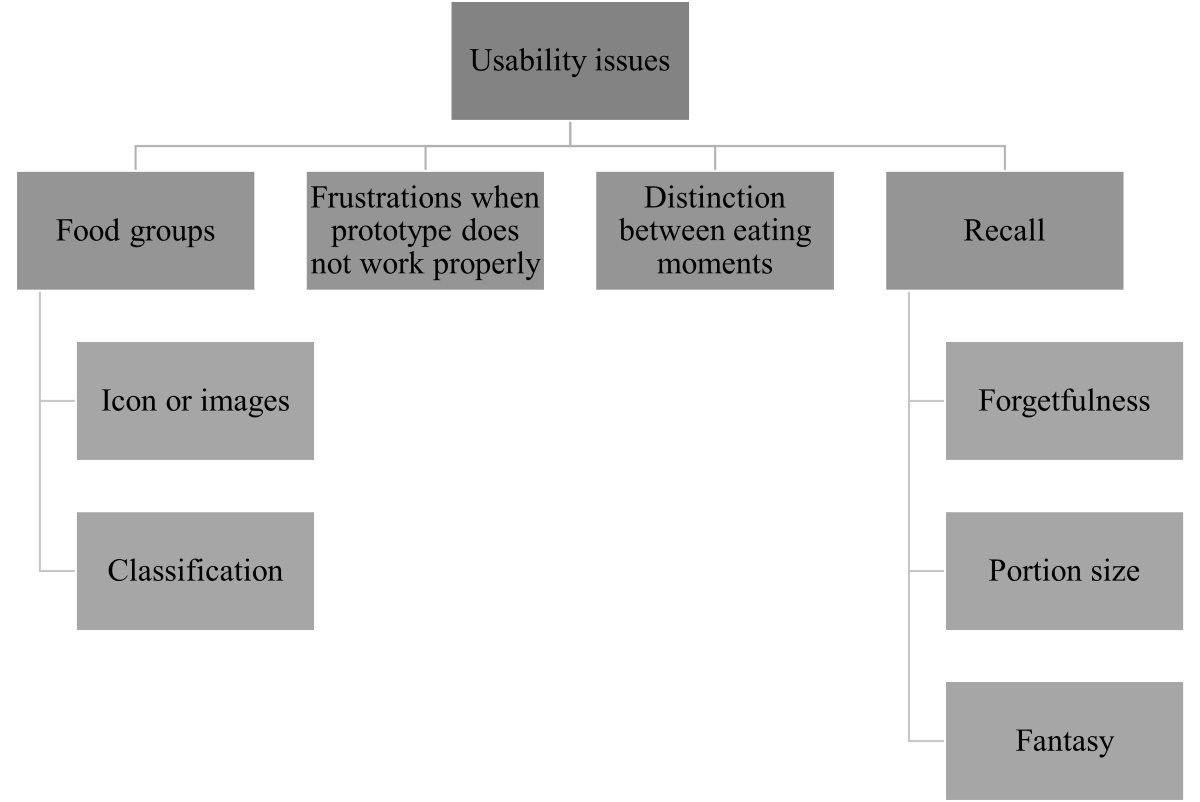
Thematic map of usability issues revealed by user testing of the dietary assessment tool prototypes FoodBear, myBear, and FoodCam.

Thematic analysis of the video and field notes obtained during usability testing revealed several key usability issues that were mainly related to 4 themes: *food groups*, *frustrations related to unsatisfactory functioning of (parts of) the prototype*, *recall of food products,* and *distinction between eating moments* (Figure 3).

#### Theme 1: Food Groups

In terms of food group–related usability issues, 93% (13/14) of the children reported problems related to the *recognition of icon/images* and *classification of food products into food groups*. When using FoodBear and myBear, approximately all children (13/14, 93%) experienced usability problems related to the depicted icons or images, which were mainly related to *recognizing* the food group represented by the icon, resulting in incorrect food group reporting. To illustrate, most children (n=10, 71%) became confused when the consumed product did not exactly resemble the depicted icon: “Cheese spread and jam. Which one is the jam?” (Participant 11). Some children (n=8, 57%) solved the problem by choosing the icon that they thought most resembled the product they ate. In some cases (n=5, 36%), the children eventually classified food products into the correct food group: “I don’t see baguette, but I do see bread, so I press the bread” (Participant 10), but other participants failed to do so: “Which one is gingerbread? This one? [points to icon for fish]” (Participant 14). Moreover, 21% (3/14) of the children ate >1 product of the same food group during their lunch and reported problems with categorizing these different food products into the same food group. One participant asked the researcher for help and the other 2 chose different icons for different products. Finally, it was also observed that some icons were not chosen at all even though the icon was applicable for several of the children, for example, the icon for “something else.”

#### Theme 2: Frustrations Related to Unsatisfactory Functioning of (Parts of) the Prototypes

Overall, 93% (13/14) of the children encountered ≥1 usability problem related to unsatisfactory functioning of (parts of) the prototypes, that is, prototype-specific issues. For FoodBear, several children encountered problems with *where and how to put coins in FoodBear’s belly* (n=4, 29%) and that *coins did not fit properly* through the intended opening (n=6, 43%). The latter led to visible frustrations among participants: “Stupid bear!” (Participant 6). When using myBear, the main usability issues were caused by *interaction design*. Most children (n=9, 64%) struggled with the plus and minus button to indicate the amount of the product eaten; children either pressed the button again, causing the product to disappear from myBear’s belly or asked the researcher for help. FoodCam’s usability issues were mainly related to *finding the photo button* (n=5, 36%) and *quality of the printed photos* (n=6, 43%): “It’s annoying that you cannot choose colors when you print it, so you can see the right colors” (Participant 6).

#### Theme 3: Distinction Between Eating Moments

More than half of the participants (n=8, 57%) had difficulties with *distinguishing eating moments throughout the day*. Some children (n=4, 29%) pointed out that they did not fully understand the concept of lunch. Other children (n=2, 14%) tended to also name products they consumed earlier that day: “But I had also yogurt today!” (Participant 6).

#### Theme 4: Recall

In the category of recall-related usability issues, the subcategories *forgetfulness*, *portion size*, and *fantasy* issues were distinguished. Overall, 57% (8/14) of the children reported usability issues related to ≥1 of these subcategories. For FoodBear and myBear, 29% (4/14) of the children failed to perform the usability tasks because of *forgetfulness*. For example, children forgot to feed certain products to myBear or FoodBear: “O, I just completely forgot about that one!” (Participant 6). Moreover, 36% (5/14) of the children had problems indicating consumed *quantities*, which resulted in underreporting by all 5 children, that is, an insufficient number of icons or coins for a specific food group in FoodBear’s or myBear’s belly. Finally, 21% (3/14) of the children were unable to complete the usability tasks because of their *fantasy*, that is, children fed FoodBear or myBear products that were not consumed (n=2, 14%) or expressed unrealistic amounts (n=1, 7%). To illustrate the latter, one participant reported: “I think ten breads. In every lunchbox” (Participant 2).

### User Experience

#### Quantitative Results

No significant difference in mean preference scores for FoodBear, myBear, and FoodCam was observed based on the This or That method, that is, 0.57 (SD 1.94), 0.50 (SD 1.45), and 0.86 (SD 1.65; *F*_2_=0.18; *P*=.80), respectively (Table 4).

**Table 4:** Results after applying This or That method. The first choice is defined as the prototype that children selected as their top preference (ie, most points). Conversely, the last choice defined is defined as the prototype they ranked as the least preferred option (ie, the least points).

	FoodBear	myBear	FoodCam
First choice, n (%)^a^	5 (36)	5 (36)	6 (43)
Last choice, n (%)^a^	7 (50)	7 (50)	5 (36)
Score, mean (SD)	0.57 (1.94)	0.50 (1.45)	0.86 (1.65)

^a^Percentages are calculated based on the number of times participants selected each prototype. As children were allowed to choose more than one prototype, the sum of percentages may exceed 100%.

#### Observational Results

During the behavioral choice selection (Textbox 2; step 9), all children (14/14, 100%) chose (one of) the prototypes they scored best during This or That.

#### Qualitative Results

Although some children (n=5, 36%) experienced difficulties with answering the “why-questions” that followed the 5 This or That questions, several determinants for product liking could be identified. First, an important reason for children to choose one prototype over the others was *autonomy*, because they liked being able to do it “themselves” (n=5, 36%): “Because I can put the coins in there myself!” (Participant 13). Moreover, children (n=5, 36%) referred to the *reward*, such as the printed photo, as being a determinant for product liking: “I like that one because you can print your taken picture and keep it as a memory!” (Participant 6). In addition, 29% (4/14) of the children indicated that they liked the prototype being *challenging*: *“*I like that one because you can do a lot with that one. The camera is a bit stupid because you can only take a picture with it” (Participant 4). Such a challenge could be presented in the form of a game; the *gaming element* was emphasized by some children (n=4, 29%) as a fun element of one of the prototypes: “I like myBear because you can play games on it” (Participant 9). The shape of the prototype was also mentioned by several children; 29% (4/14) of the children indicated that they liked the prototype because it was *tablet based* or because they liked its *appearance* (n=2, 14%). Other determinants of preference included its *social aspect* (n=2, 14%) and the *time frame* (n=2, 14%): “I didn’t like this one very much because this one took too long and that one took too short” (Participant 13).

## Discussion

### Principal Findings

This study provided several insights related to usability and user experience that can be used to inform the development of dietary assessment tools for use by children. At the first encounter, most children were able to use FoodBear, myBear, and FoodCam and fulfill the accompanying usability tasks. However, all children required assistance from the researcher to succeed, and most of the children encountered several usability problems. The most important usability issues included problems related to food groups, frustrations related to the unsatisfactory functioning of (parts of) the prototypes, recall of food products, and distinction between eating moments. These issues, along with the queries needed to accomplish usability tasks, may suggest that dietary assessment tools may not be independently usable by children aged 5 to 6 years. However, the completion rates suggest that children can play a complementary role in dietary data collection to enhance data collected by their parents. No differences in product liking were observed when comparing the 3 prototypes. However, it is notable that all children selected one of the prototypes they scored best with This or That for the behavioral choice selection to play with again. The qualitative part of This or That revealed several determinants for liking a product, including autonomy, challenge, gaming elements, being tablet based, appearance, social elements, and time frame.

### Usability

#### Overview

The 3 prototypes differed in terms of usability rate, time needed to complete the assessment, and required number of interruptions by the researcher, which may be partly explained by the fact that prototypes are based on different dietary assessment methodologies, that is, food recall (FoodBear and myBear) and food record (FoodCam) [9]. As a recall requires additional memory-based cognitive capacities compared with a food record, FoodCam was expected to yield the best results in this population. However, a lower number of children successfully completed FoodCam’s (9/14, 64%) usability task compared with FoodBear’s (11/14, 79%) and myBear’s (10/14, 71%) tasks, meaning that a lower number of children were able to take a sharp photo on which all consumed products were recognizable. On the other hand, if completed, the time and help needed with FoodCam’s usability task was substantially less than the other 2 tools. Aflague et al [27] showed that using the Mobile Food Record for capturing eating occasions could be a feasible method for use by children aged ≥3 years. In contrast to our study, participants in the study by Aflague et al [27] were allowed to practice and use a tablet or smartphone, whereas FoodCam is based on a more old-fashioned camera. Additional research is required to determine whether there is a difference in usability between traditional cameras and the cameras on smartphones or tablets. However, this research should also address the current challenges related to automatically extracting dietary information from real-world, user-generated images. As our prototypes were tested at the first encounter, it is likely that usability will increase with practice or a training module, but further studies are required to test this. Moreover, this study primarily evaluated the usability of 3 prototypes designed for independent use, thereby revealing some inherent challenges. Nonetheless, adopting an approach that combines children’s data with those collected by parents can potentially enrich the comprehensiveness of a child’s daily dietary intake assessment. Such a combined method would offer the possibility of gaining more detailed insights into foods consumed outside the home, ultimately enhancing the reliability of the dietary intake data. Further research is needed to investigate the potential bias of this approach. Moreover, consistent with previous findings [23,35], girls performed better than boys for all 3 usability and lunch recall tasks in terms of completion rate. This difference may be explained by girls’ higher attentional and memory performance compared with boys and emphasizes the need to consider sex differences in further development of the tools [36].

#### Strategies to Increase Usability

To address these usability issues, we identified several strategies for further improvement. To increase usability, the tools might benefit from a *training* *module* providing practice runs on estimating quantities and portion sizes, recognizing food categories, handling FoodCam, or the interaction design of myBear. Practical effects were already observed in this study, that is, all children who successfully completed myBear’s or FoodBear’s usability task performed their second usability task at a faster pace. Similar training effects have been observed in other studies [27,37]. *Auditory or visual prompts, reminders, and feedback* may also improve the usability of updated versions of the prototypes, that is, to remind participants to report their dietary intake throughout the day, or help with the correct use of the tool, for example, by checking whether all products have been reported in the correct amount, or send reminders when photos are incomplete or unsharp. Reminders and help with the tasks were now verbally performed by the researcher (eg, with recall questions), but should be automated in the next versions of the prototypes to facilitate independent use by the target group. Integrating multiple reminders is commonly used in other methods as well, for example, in Compl-eat [38], and is used to trigger the report of often forgotten products, such as cooking fats or drinks. In addition, myBear could particularly benefit from a more user-friendly and intuitive interaction design. Improvements in the interaction design should among others focus on simplifying consumed portion sizes. For example, using a slider to indicate portion size or pressing the button twice for the specific product may be more intuitive than using a plus or minus button. The use of age-appropriate interactions and images could also contribute to a better understanding of the different eating moments, for example, by using a clock model to capture mealtimes throughout the day. The direct effect of improving the interaction design has proven to be effective in another study on the adolescent dietary assessment tool myfood24, where they compared the usability and acceptability of myfood24 among adolescents before and after making amendments [39]. Finally, the usability tasks of FoodBear and myBear illustrated that children experienced difficulties in understanding or interpreting the food group icons and categorizing their consumed products into food groups, asking for a more child-friendly approach. Therefore, further research is needed to identify *child-friendly food groups and icons* [25].

### User Experience

No preferences were observed for one prototype over the others when using This or That method. However, it is notable that all children selected one of the prototypes they scored best with This or That to play again. This consistency suggests that the reported This or That choice is a good predictor for short-term preference in this sample. However, as the children only used the prototypes for a short time, it should be emphasized that This or That may not reflect the long-term preference. To gain insight into long-term engagement, more research is needed where children use the prototypes for a longer period in a home-use setting handling different mealtimes throughout the day.

As This or That determines preference relatively, it does not offer the opportunity to determine the magnitude of preference [34]. Although the This or That scores revealed no differences between the prototypes, it is important to consider this relativity when interpreting the qualitative results. What stands out is that most of the determinants for product liking pointed out by the children were in line with our list of requirements (Textbox 1), except for the determinant autonomy. Children in the preoperational phase, including our target group, have a strong curiosity and are interested in learning [40]. Therefore, the finding of autonomy being a determinant for product liking by children is not unexpected and should be included in the updated version of the program of requirements. Strategies to increase this feeling of autonomy within young children’s dietary assessment could include, for example, *making the design accessible for children’s independent navigation* (eg, by using navigation without text and making it real-time responsive), *focus on children’s decision-making* (eg, by including options for personalization and customization in the design), or *encouraging their initiatives* (eg, by including a reward system) [41].

### Strengths and Limitations

Although this exploratory study contributes to the body of knowledge in several areas, it has some limitations. First, our first list of user requirements (Textbox 1) is based on literature only, which ideally would have included expert interviews as well, as conducted by de Gooijer et al [42]. As this study is the first to explore self-reported dietary assessment among children aged 5 to 6 years, this first list may have been insufficient. Second, as the usability tasks in this study were performed under favorable circumstances, the usability for FoodBear and myBear may have been overestimated. More specifically, the dietary assessment tasks took place shortly after lunch (a maximum of 3 hours after lunch). As other studies showed that meals with shorter retention intervals are in general easier to accurately recall and report compared with meals with longer retention intervals, results may become less accurate when measurements were performed after a longer period [43,44]. Moreover, prototypes were only evaluated for lunch and longer interaction (eg, over the course of a day) with the prototypes is needed to evaluate the accuracy of dietary intake data collected through our prototypes. In addition, it is worth noting that Dutch children typically have bread with spreads or toppings for lunch [45]. This was also reflected in our study sample, where all participating children ate bread for lunch. As previous studies showed that children struggle with identifying components within mixed meals [46], it is important to consider the relative simplicity of the Dutch lunch when interpreting our results. Considering these favorable conditions in this study, it raises questions about the ability of children aged 5 to 6 years to accurately use FoodBear and myBear without parental assistance for more complex meals consumed over an extended time frame in future research. Therefore, FoodBear and myBear might have more potential for use with caregivers. Another important limitation that should be considered when interpreting our findings is related to our sample. The sample size of this study was small and not representative of the Dutch population, primarily owing to the high proportion of highly educated parents among the participants. This demographic bias limits our ability to generalize our findings to a more diverse population. To gain a better understanding of the application of such tools in populations of lower socioeconomic status, further studies are necessary. Moreover, considering the qualitative focus of our research, the small sample size of our study underscores the necessity for caution when interpreting our quantitative results. This is particularly relevant in terms of statistical power and generalizability. Future research efforts could focus on studying the quantitative aspects of our study in more detail by recruiting a larger sample size.

### Conclusions

This exploratory study identified essential user requirements for a novel dietary assessment tool designed for children aged 5 to 6 years, including (1) a comprehensive training program, (2) incorporation of auditory or visual prompts, (3) implementing reminders and feedback mechanisms, (4) a focus on a user-friendly and intuitive interaction design, (5) use of child-friendly food groups or icons, and (6) allowing room for children to exercise autonomy. By addressing these identified user requirements in the development of new dietary assessment tools, we can significantly enhance the quality of dietary intake data collected among children. Furthermore, these findings can serve as valuable guidance for ongoing innovations in the field of children’s dietary assessment and the provision of personalized dietary support. This, in turn, can inform strategies aimed at guiding children toward healthier food choices.

## Appendix

Multimedia Appendix 1 Summary of weighted decision matrixes.

Multimedia Appendix 2 The functionality of myBear and FoodCam.

## Abbreviations

WDM: weighted decision matrix
WUR: Wageningen University and Research

## Data Availability

The data sets generated during or analyzed during this study are available from the corresponding author upon reasonable request.
