# Supplementary material for: User Requirements in Developing a Novel Dietary Assessment Tool for Children: Mixed Methods Study
Source: JMIR Form Res. 2024 Feb 1;8:e47850. doi: 10.2196/47850 (PMC10870213; doi:10.2196/47850)
Supplement: Multimedia Appendix 1 [file formative_v8i1e47850_app1.docx]

|  |  | Concepts | | | | | | | | | | | |
| --- | --- | --- | --- | --- | --- | --- | --- | --- | --- | --- | --- | --- | --- |
| Requirements | **Weight** | **FoodBear** | | **FoodCam** | | **KidsPlate** | | **Tamagotchi** | | **Smartwatch** | | **FoodDiary** | |
|  |  | **Score** | **Total** | **Score** | **Total** | **Score** | **Total** | **Score** | **Total** | **Score** | **Total** | **Score** | **Total** |
| Collect accurate and useful data on dietary intake in children | 5 | 5 | 25 | 5 | 25 | 5 | 25 | 5 | 25 | 5 | 25 | 5 | 25 |
| Be understandable (i.e., simple, easy-to-use and intuitive) | 5 | 5 | 25 | 3.7 | 18.5 | 3.3 | 16.5 | 3.7 | 18.5 | 3.7 | 18.5 | 4 | 20 |
| Be fast paced (i.e., completed in a short time) | 5 | 4.7 | 23.5 | 4 | 20 | 3.3 | 16.5 | 3.3 | 16.5 | 4.3 | 21.5 | 2.7 | 13.5 |
| Give feedback and context-specific help, (e.g., auditory or visual feedback) | 4 | 4 | 16 | 4.7 | 20 | 3.7 | 14.8 | 5 | 20 | 4.7 | 18.8 | 1.3 | 5.2 |
| Be motivating or encouraging to use | 4 | 4.3 | 17.3 | 3.7 | 14.8 | 2.3 | 9.2 | 5 | 20 | 3 | 12 | 2.7 | 10.8 |
| Be social | 3 | 3.3 | 9.9 | 3 | 9 | 1.7 | 5.1 | 5 | 15 | 2.7 | 8.1 | 3.7 | 11.1 |
| Be challenging | 3 | 2.7 | 8.1 | 4.7 | 14.1 | 3 | 9 | 3.7 | 11.1 | 4.3 | 12.9 | 3 | 9 |
| Wishes |  |  | | | | | | | | | | | |
| Incorporate photography | 1 | 1 | 1 | 5 | 5 | 1 | 1 | 1 | 1 | 3.7 | 3.7 | 5 | 5 |
| Incorporating an avatar | 1 | 4.3 | 4.3 | 5 | 5 | 1 | 1 | 5 | 5 | 2 | 2 | 1 | 1 |
| Incorporate gamification | 1 | 3 | 3 | 2 | 2 | 2.3 | 2.3 | 4.3 | 4.3 | 2 | 2 | 2 | 2 |
| Include a storyline | 1 | 2.7 | 2.7 | 1.3 | 1.3 | 1.3 | 1.3 | 3 | 3 | 1.3 | 1.3 | 1 | 1 |
| Include rewards | 1 | 4 | 4 | 5 | 5 | 2.3 | 2.3 | 5 | 5 | 5 | 5 | 1 | 1 |
| Incorporate learning and/or repetitive elements | 1 | 4.3 | 4.3 | 5 | 5 | 4.3 | 4.3 | 4 | 4 | 3.3 | 3.3 | 4 | 4 |
|  |  |  | | | | | | | | | | | |
|  | Total: |  | 144.1 |  | 144.7 |  | 107.3 |  | 148.4 |  | 134.1 |  | 108.6 |

**Summary Weighted Decision Matrixes**
