# Supplementary material for: User Requirements in Developing a Novel Dietary Assessment Tool for Children: Mixed Methods Study
Source: JMIR Form Res. 2024 Feb 1;8:e47850. doi: 10.2196/47850 (PMC10870213; doi:10.2196/47850)
Supplement: Multimedia Appendix 2 [file formative_v8i1e47850_app2.docx]

**Functionality of myBear**

Screenshots of myBear

#
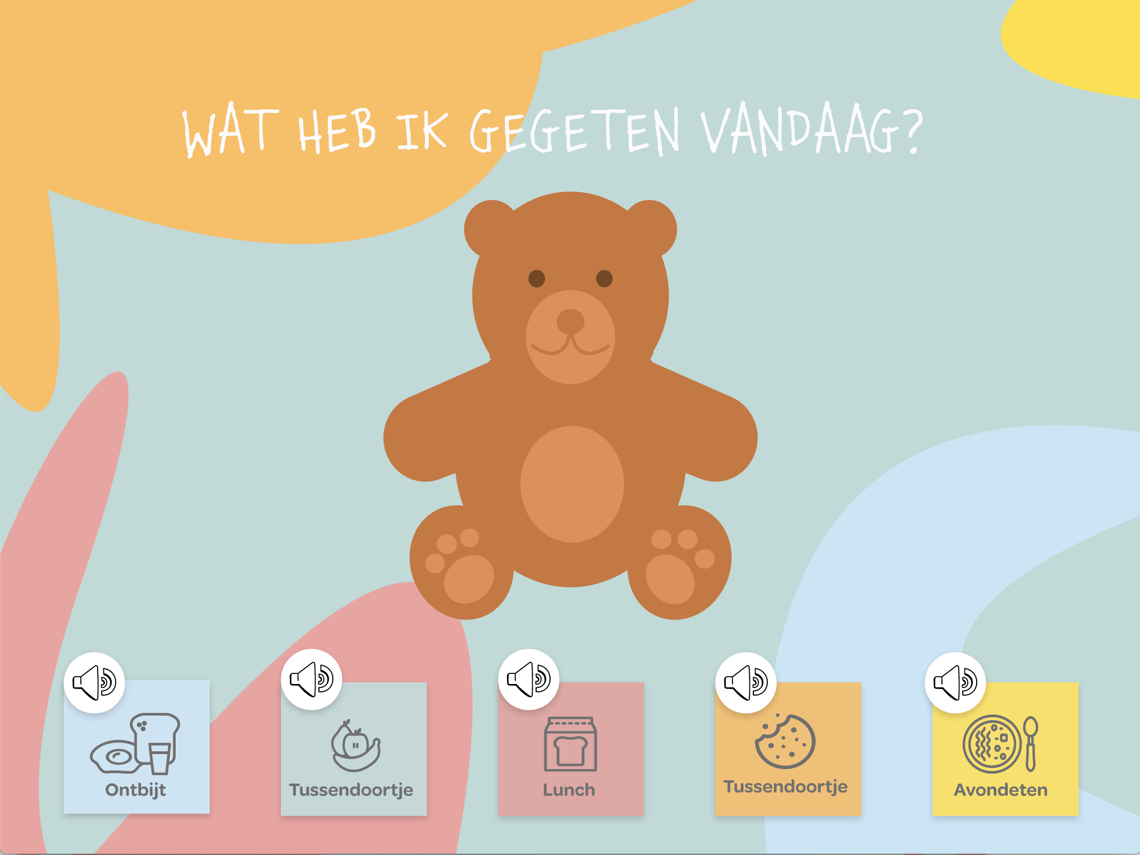

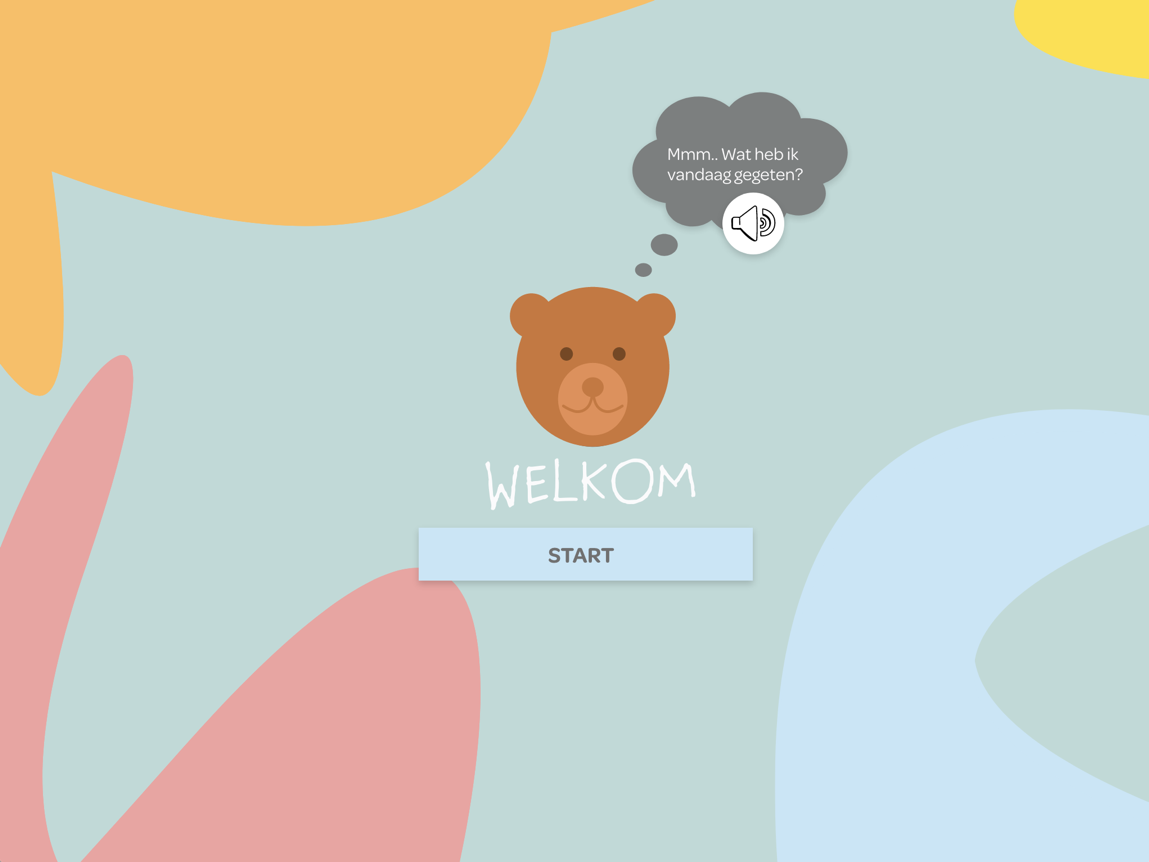


Figure 2: Screenshot main screen myBear


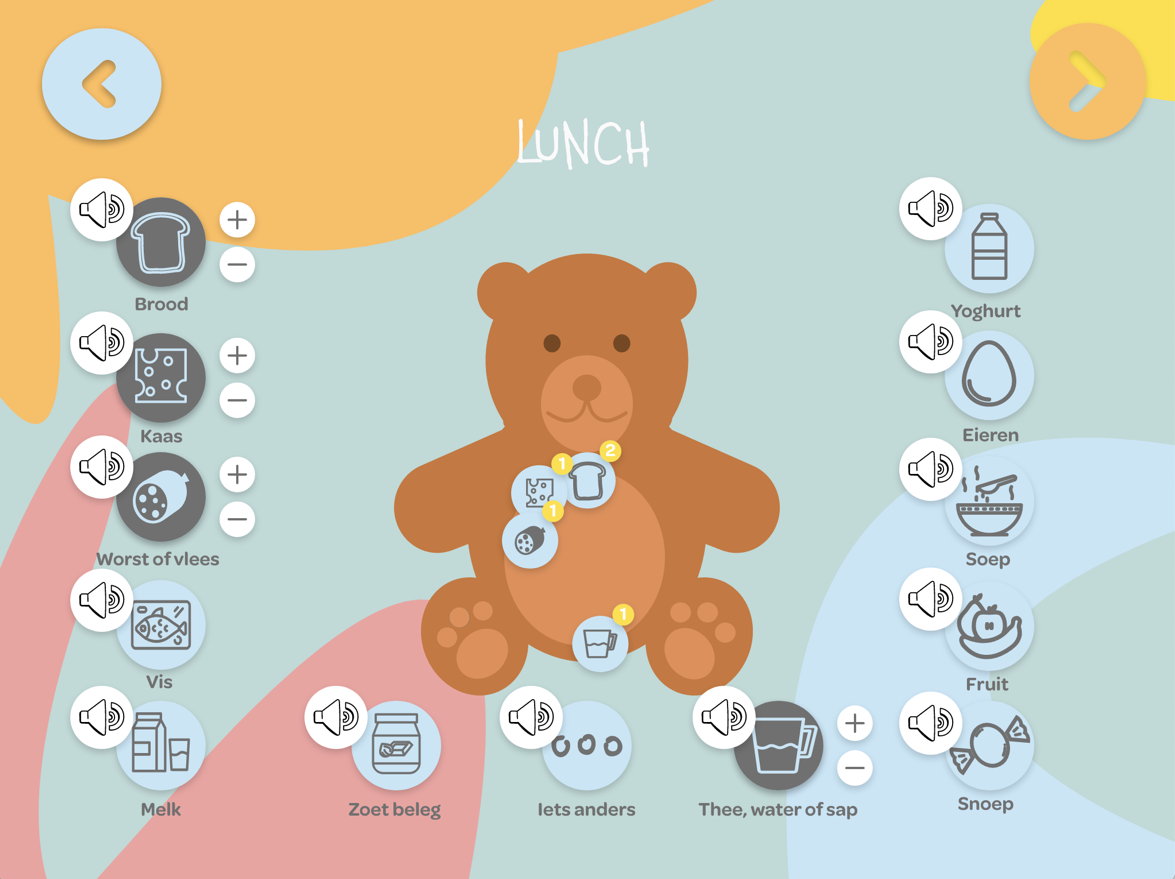

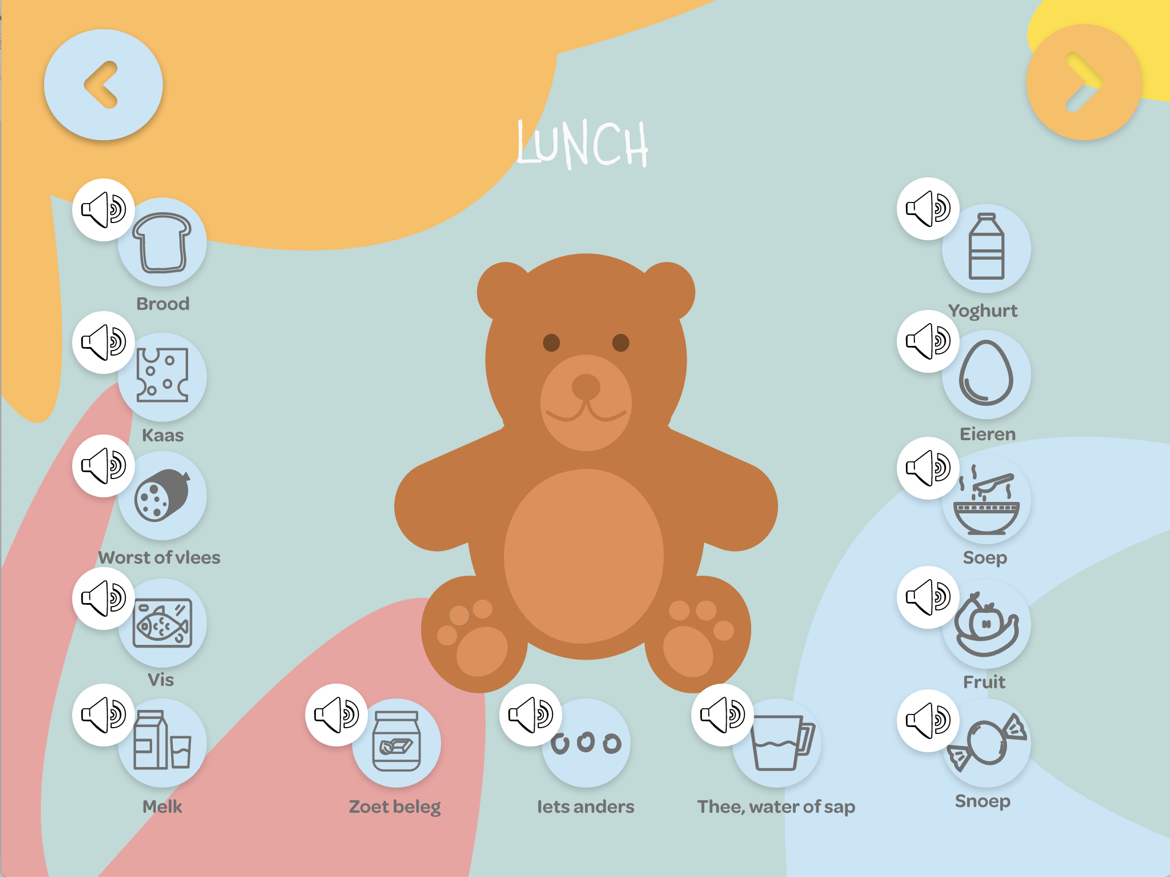

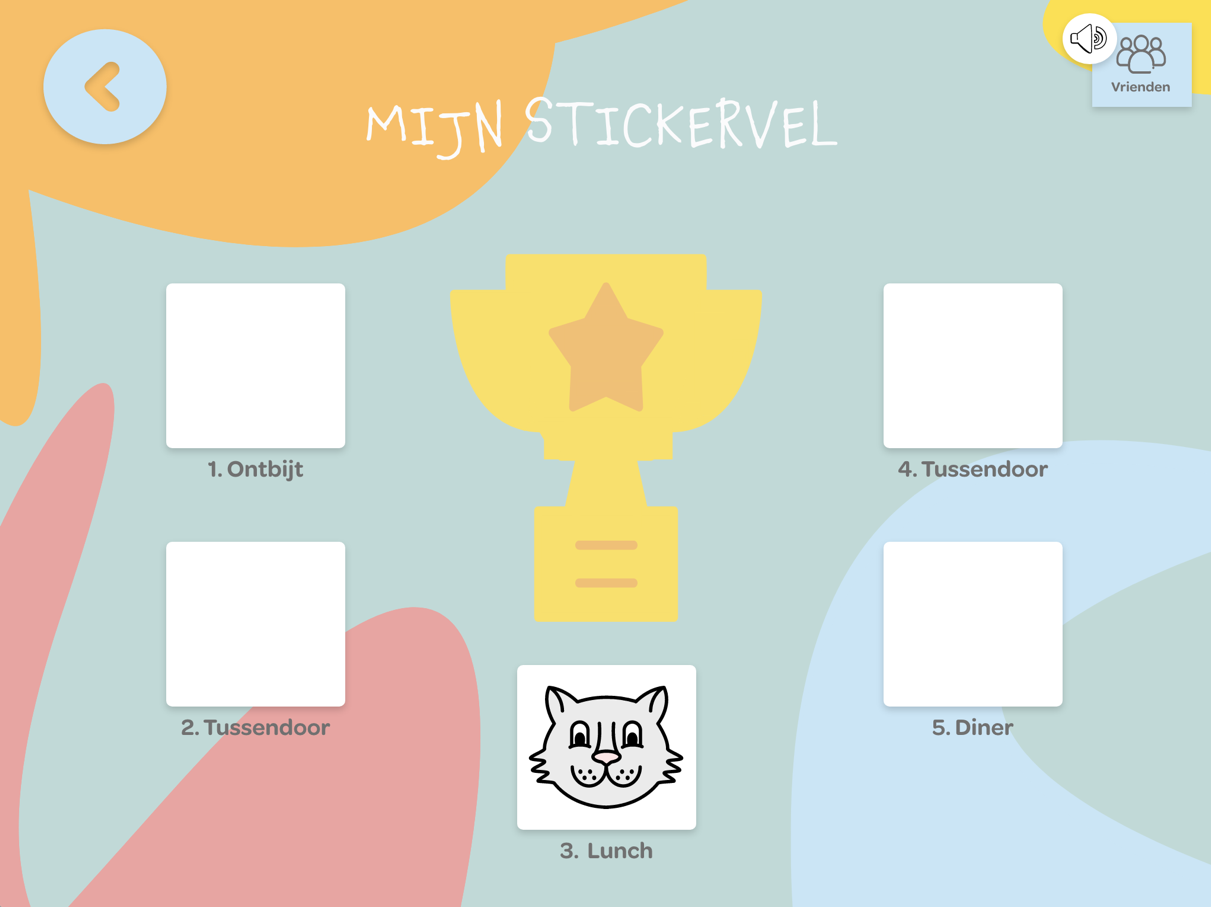

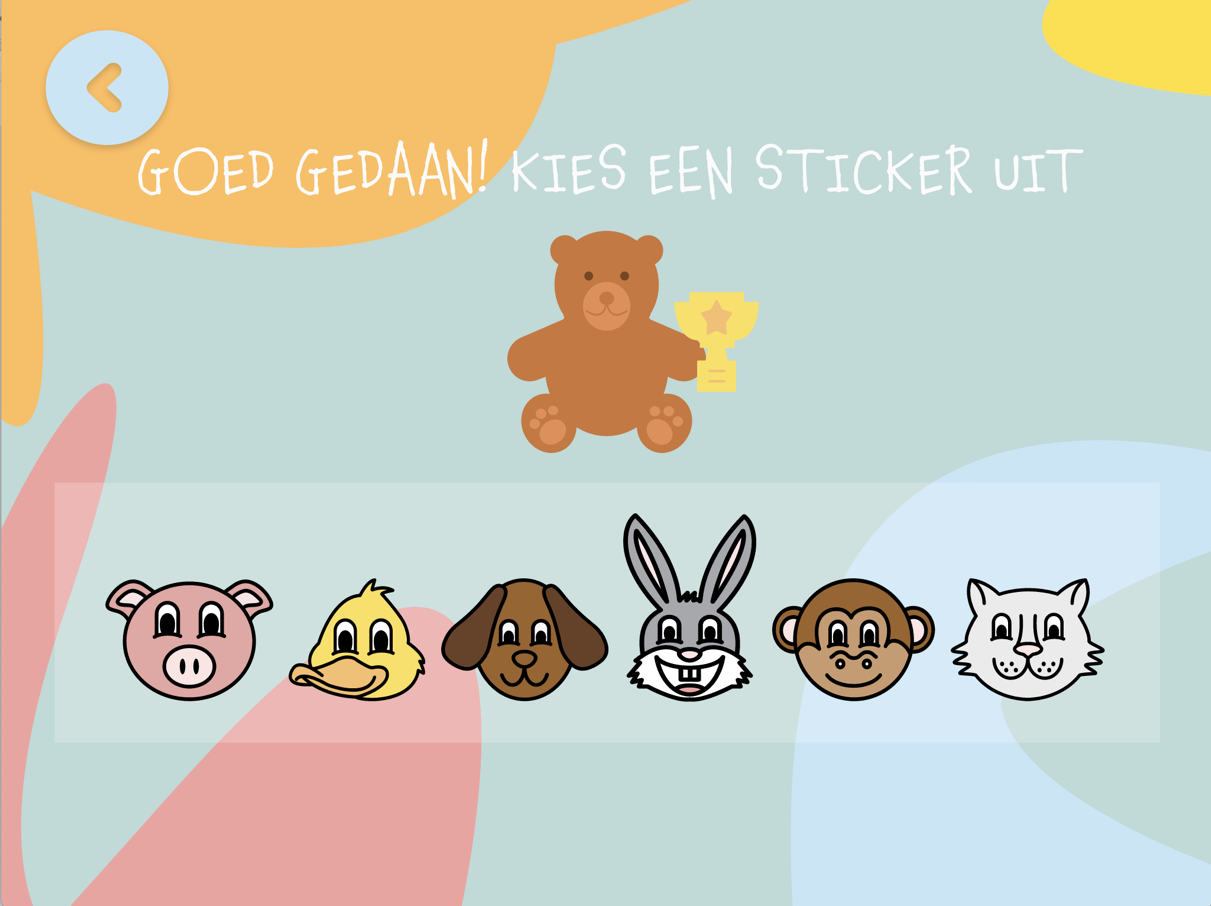


Figure 1: Screenshot startscreen myBear

Figure 4: Screenshot example lunch myBear

Figure 3: Screenshot screen lunch myBear

Figure 8: Screenshot of the sticker sheet-screen myBear. In this example, the child chose the sticker of the cat.

Figure 5: Screenshot of the sticker-screen myBear.
